# Performance of 2 Commercial Serologic Tests for Diagnosing Zika Virus Infection

**DOI:** 10.3201/eid2506.180361

**Published:** 2019-06

**Authors:** Séverine Matheus, Cheikh Talla, Bhety Labeau, Franck de Laval, Sébastien Briolant, Lena Berthelot, Muriel Vray, Dominique Rousset

**Affiliations:** Institut Pasteur de la Guyane, Cayenne, French Guiana (S. Matheus, B. Labeau, L. Berthelot, D. Rousset);; Institut Pasteur de Dakar, Dakar, Senegal (C. Talla, M. Vray);; French Armed Forces Health Service in French Guiana, Cayenne (F. de Laval);; French Military Centre for Epidemiology and Public Health, Marseille, France (F. de Laval);; Institut de Recherche Biomédicale des Armées, Marseille (S. Briolant); Aix Marseille Université, Marseille (S. Briolant)

**Keywords:** Zika virus, serology, sensitivity, specificity, cross reactions, antibody kinetics, French Guiana, diagnosis, viruses

## Abstract

Reliable serologic tests are needed for diagnosis and surveillance of Zika virus infection. We evaluated the Euroimmun and Dia.Pro serologic tests for detection of Zika virus IgM and IgG by using a panel of 199 samples from a region endemic for flaviviruses. Kinetics of Zika virus antibodies were monitored from 300 sequential specimens sampled over a period of 10 months after infection. We observed suboptimal performance; sensitivity for Zika virus IgM was low, especially in the Euroimmun assay (49%), whereas IgM could be detected for months with the Dia.pro assay. The specificity of the Zika virus IgG assays was also low, especially that of Dia.Pro (62%); findings were strongly influenced by the epidemiologic context. These results highlight the complexity of serologic diagnosis of Zika virus infection in regions endemic for flaviviruses. Accurate analysis of the performance of assays is required to adapt and interpret algorithms.

Zika virus belongs to the Flaviviridae family, genus *Flavivirus*, and is an arbovirus transmitted mainly by mosquitoes of the genus *Aedes* (*Stegomyia*). Initially isolated in 1947 from a sentinel monkey during yellow fever surveillance in Uganda, Zika virus was reported as causing only sporadic human infections, associated with asymptomatic or mild, self-limiting illness, until 2007 (*1**,**2*). In 2007, Zika virus spread first to Pacific islands and then throughout the Americas, resulting in large outbreaks in several regions of the world. Zika virus infection is estimated to be symptomatic in 18%–73% of cases (*3**–**5*); severe complications have been reported, including neurologic disorders, such as Guillain-Barré syndrome, and congenital Zika virus syndrome, which is characterized by severe microcephaly, brain and ocular anomalies, congenital contractures, and neurologic impairment in the fetuses and newborns of mothers infected during pregnancy (*3**,**4**,**6*).

The mild signs and symptoms of Zika virus infection include fever, rash, joint pain, conjunctivitis, headache, and myalgia (*7*)*.* These manifestations are difficult to distinguish clinically from those caused by other arboviral infections, such as dengue or chikungunya, which are often observed in the same geographic areas. Therefore, specific, reliable diagnostic tools are needed.

Several commercial kits are available for direct viral detection by nucleic acid–based testing, which enable diagnosis during the acute phase of the disease (*8**,**9*): up to 7 days after symptom onset in serum samples, up to 20 days in urine, and even longer in semen (*10**–**13*). This virologic window, combined with the high proportion of asymptomatic forms, makes the monitoring of Zika virus infection difficult, especially in pregnant women. Therefore, serologic tools for diagnosis of Zika virus infection are urgently needed. This challenge remains because of cross-reactivity among flaviviruses, especially in a context of secondary flavivirus infection or previous immunization.

The first objective of this study was to evaluate the performance of two commercial serologic kits for the detection of Zika virus–specific IgM and IgG in serum samples from patients with an arboviral-like syndrome in a region where other arboviruses are known to circulate, including dengue and chikungunya viruses. The 2 commercial kits (4 assays) studied were the Euroimmun Zika virus IgM and IgG ELISAs (https://www.euroimmun.com) and Dia.Pro Zika virus IgM and IgG ELISAs (Diagnostic Bioprobes Srl, https://www.diapro.it). The second objective was to determine the kinetics of Zika virus IgM and IgG induced after infection, as defined by these kits.

## Material and Methods

### Clinical Samples and Study Design

The clinical samples used in this study were selected according to the standards for reporting diagnostic accuracy requirements. Two thirds came from the serum collection of the National Reference Centre (NRC) for Arboviruses in French Guiana and one third from samples collected for a descriptive prospective study of Zika virus disease in the French military community in French Guiana (ZIFAG) (*14*). The NRC collection comprises clinical specimens received during 2002–2017 for routine diagnosis and expertise. The protocol of the ZIFAG study received ethics approval from the Comité de Protection des Personnes Sud-Méditerranée I (ID RCB: 2016-A00394–47); all participants provided written informed consent. All the selected specimens were obtained from patients with an arboviral-like syndrome and an etiologic diagnosis confirmed by real-time reverse transcription PCR (RT-PCR) on acute-phase serum or urine samples. A commercial qualitative RT-PCR kit (Altona Diagnostics, https://www.altona-diagnostics.com) was used for Zika virus detection and NRC in-house real-time RT-PCR for dengue and chikungunya viruses.

We first evaluated the performance of the 2 commercial immunoassays against a panel of 199 serum samples collected from days 3 through 20 after the onset of symptoms (onset defined as day 0—that is, within the first 24 hours) (Table 1). We evaluated the sensitivity of the assays in a subgroup of 90 serum samples from 90 patients with confirmed Zika virus infection diagnosed from the end of 2015 through 2016, during the outbreak in French Guiana (Zika subgroup). We evaluated the specificity of the assays in a subgroup of 109 serum samples with a strong potential for causing flavivirus cross-reactions (non-Zika subgroup). This subgroup comprised serum samples obtained from 35 patients with confirmed dengue virus infection sampled during 2002–2013 dengue epidemics; 29 patients with confirmed chikungunya virus infection sampled during the chikungunya outbreak in French Guiana in 2014–2015, just after the dengue outbreak in 2012–2013; and 45 patients with neither dengue virus, chikungunya virus, nor Zika virus infection sampled just before the Zika virus outbreak. We peformed Zika microneutralization tests on the Zika virus, dengue virus, and chikungunya virus–negative subgroup of serum samples, enabling confirmation of the absence of Zika neutralizing antibodies.

**Table 1:** Characteristics of panels evaluated in study of Zika virus diagnostic tests

Panel	Sample characterization	No. samples	Mean time to collection after onset of fever, d (+ SD)
Panel performance evaluation 3–20 days after onset			
Zika virus subgroup	Positive Zika virus	90	10 + 6
Non–Zika virus subgroup	Negative Zika virus with confirmed dengue virus infection	35	10 + 3
	Negative Zika virus with confirmed chikungunya virus infection	29	13 + 3
	Negative Zika virus, dengue virus, chikungunya virus	45	11 + 5
Total panel performance		199	10 + 5
Panel IgM–IgG kinetics 0–300 days after onset			
Positive Zika virus		300*	Minimum 0, maximum 300, median 20, interquartile range 4–81

*300 samples from 124 patients, including the 90 positive Zika virus samples in the first panel.

To determine the kinetics of Zika virus antibodies, we used a panel of 300 serum samples collected from 124 patients with confirmed Zika virus infection from day 0 through day 300 after the onset of symptoms (Table 1). We collected 1–8 samples from each Zika virus–infected patient. The distribution of samples according to time since onset was as follows: 76 samples collected during days 0–4, 50 during days 5–14, 55 during days 15–30, 19 during days 31–60, 32 during days 61–90, 44 during days 91–180, and 24 during days 181–300.

### Euroimmun and Dia.Pro Zika Virus IgM and IgG ELISAs

We tested all samples with the Euroimmun ELISAs according to the manufacturer’s recommendations and calculated signal-to-cutoff (S/CO) ratios; values <0.8 were regarded as negative, >0.8 to <1.1 as equivocal, and >1.1 as positive. We tested all serum specimens with the respective Dia.Pro ELISAs for qualitative determination of IgM and IgG, according to the manufacturer’s instructions; we interpreted results as positive if the S/CO ratio was >1.1, negative if <0.9, and equivocal if 0.9–1.1. Recombinant Zika virus nonstructural protein 1 was the antigen in both the Euroimmun and Dia.Pro assays.

### Statistical Analysis

Continuous variables were expressed as mean (± SD) or median with interquartile range (IQR) and discrete variables as percentages and 95% CIs. We calculated the sensitivity and specificity of the assays with 95% CIs. The differences in the S/CO ratios of the IgM and IgG assays at each time were compared with the Student *t* test. We performed all statistical analyses using R 3.4 statistical software (https://www.r-project.org).

## Results

### Patient Characteristics

The 199 samples used to evaluate the performance of the serologic kits came from patients with a mean + SD age of 36 + 16 years (range 1–74 years; IQR 27–46 years). This panel was composed of the Zika virus–positive subgroup (n = 90), 51 (57%) female and 39 (43%) male, with a mean age + SD of 39 + 12 years; and the Zika virus–negative subgroup (n = 109), 62 (57%) female and 45 (43%) male, with a mean + SD age of 34 + 18 years (Table 1).

The 300 samples used to determine the kinetics of Zika virus IgM and IgG came from 124 patients, 82 (66%) female and 42 (34%) male. The age range of these patients was 8–74 years (mean + SD 37 + 11 years).

### Performance of Zika Virus IgM ELISAs

We evaluated the sensitivity and specificity of the 2 Zika virus IgM tests against 90 Zika virus subgroup samples and 109 non-Zika subgroup samples (Table 2). Each test gave inconclusive results for 6 of the 199 samples: with the Euroimmun test, we obtained 6 inconclusive results from the 90 samples from the Zika virus subgroup, and with the Dia.Pro test, we obtained 4 inconclusive results of the 90 samples from the Zika virus subgroup and 2 for samples collected on day 9 after onset of disease from patients with confirmed dengue virus infection. 

**Table 2:** Performance of Zika virus IgM and IgG assays in panels of characterized samples obtained during days 3–20 after onset of symptoms*

Results	Zika subgroup	Non–Zika subgroup				Total	Sensitivity (95% CI), specificity (95% CI)
		All	Zika–/DENV+	Zika–/CHIKV+	Zika–/DENV–/CHIKV–		
Euroimmun IgM test							
Positive	41	1	0	0	1	42	49% (38–60), 99% (97–100)
Negative	43	108	35	29	44	151	
Inconclusive	6	0	0	0	0	6	
Total	90	109	35	29	45	199	
Dia.Pro IgM test							
Positive	59	4	3	1	0	63	69% (59–79), 96% (92–100)
Negative	27	103	30	28	45	130	
Inconclusive	4	2	2	0	0	6	
Total	90	109	35	29	45	199	
Euroimmun IgG test							
Positive	58	30	14	11	5	88	71% (61–81), 70% (61–79)
Negative	24	71	21	13	37	95	
Inconclusive	8	8	0	5	3	16	
Total	90	109	35	29	45	199	
Dia.Pro IgG test							
Positive	67	40	14	17	9	107	79% (70–88), 62% (53–71)
Negative	18	66	21	12	33	84	
Inconclusive	5	3	0	0	3	8	
Total	90	109	35	29	45	199	

*CHIKV, chikungunya virus; DENV, dengue virus; +, positive; –, negative.

The sensitivity of the Euroimmun Zika virus IgM test was 49% (41/84; 95% CI 38%–60%); sensitivity of the Dia.Pro test was 69% (59/86; 95% CI 59%–79%). Only 1 of the 109 non-Zika subgroup serum samples was positive in the Euroimmun test, indicating 99% specificity (95% CI 97%–100%). Four non-Zika subgroup samples were detected as positive by the Dia.Pro IgM test, including 3 samples from patients with acute dengue virus infection, indicating a specificity of 96% (103/107; 95% CI 92%–100%).

### Performance of Zika Virus IgG ELISAs

We used the same panel to evaluate the performance of Euroimmun and Dia.Pro Zika virus IgG kits (Table 2). With the Euroimmun test, 16 (8%) of the 199 samples gave inconclusive results, 8 among positive Zika virus samples collected on days 5 (1 sample) and 9 (7 samples) of disease onset; 8 negative Zika virus samples also gave inconclusive results (5 chikungunya virus–positive samples and 3 from the group negative for dengue virus, chikungunya virus, and Zika virus). The sensitivity of this assay was 71% (58/82; 95% CI 92%–100%) and the specificity 70% (71/101; 95% CI 61%–79%). With the Dia.Pro IgG test, 8 samples gave inconclusive results: 5 samples from the Zika virus–positive group (2 collected before day 5 and the others on day 9 or later after the onset of illness) and 3 samples from the Zika virus–negative group (collected on days 4 to 6 after onset) (Table 2). The sensitivity of this assay was 79% (67/85; 95% CI 70%–88%) and the specificity 62% (66/106; 95% CI 53%–71%). 

The false positivity rate of the 2 Zika virus IgG assays varied according to the subpanel used. These rates were 40%–58.6% for positive dengue virus or chikungunya virus sample subgroups and 11.9%–21.4% for the negative dengue virus, negative chikungunya virus, and negative Zika virus sample subgroup (Table 3).

**Table 3:** Rate and ratio of false Zika virus IgG–positive samples obtained with Euroimmun and Dia.Pro Zika virus IgG assays according to the non–Zika virus sample subgroup

Test	Positive for dengue virus, collected before 2013	Positive for chikungunya virus, collected in 2014	Negative for Zika, dengue, and chikungunya viruses, collected at end of 2015
Euroimmun IgG test			
False positivity rate, % (pos/pos + neg)	40% (14/35)	45.8% (11/24)	11.9% (5/42)
IgG S/C ratio of false-positive IgG, mean (range)	3.9 (1.1–6.7)	2.0 (1.2–2.9)	2.0 (1.3−3.7)
Dia.Pro IgG test			
False positivity rate, % (pos/pos + neg)	40% (14/35)	58.6% (17/29)	21.4% (9/42)
IgG S/C ratio of false-positive IgG, mean (range)	9.2 (2.5–14.4)	3.1 (1.1–7.7)	4.3 (1.1–10.1)

*Neg, negative; pos, positive.

### Combined Performance of IgM/IgG Assays

The sensitivity of the combined Euroimmun Zika virus IgM and IgG assays was 82% (71/87; 95% CI 74%–90%) and the specificity was 69% (70/101; 95% CI 60%–78%). The sensitivity of the combined Dia.Pro Zika virus IgM and IgG assays was 87% (75/86; 95% CI 80%–94%) and specificity was 62% (66/106; 95% CI 53%–71%).

### Time-Course Analysis of Zika Virus IgM and IgG, Days 0–300 after Onset of Symptoms

**Figure 1:**
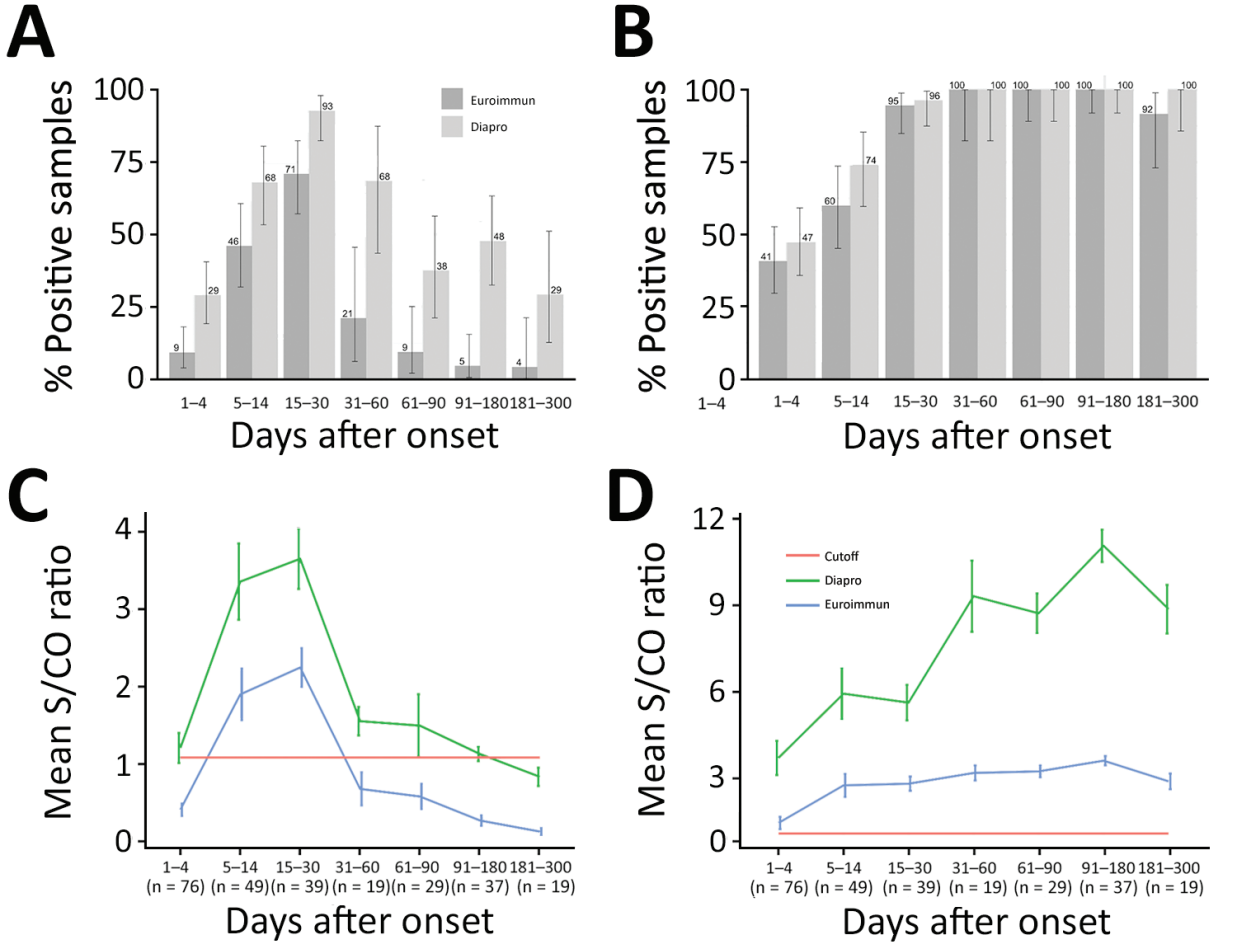
Kinetics of Zika virus IgM and IgG as determined with Euroimmun and Dia.Pro kits for patient samples collected in the first 10 months after infection, by time interval. A, B) Percent positive for Zika virus IgM (A) and IgG (B). Values are given with binomial proportion CI). C, D) Overall time course of mean signal-to-cutoff ratios of Zika virus IgM (C) and IgG (D). Values are shown with SEs. The number of patients sampled is provided for each time interval.

We used the panel of sequential samples from patients with confirmed Zika virus infection to determine the kinetics of Zika virus IgM and IgG over 10 months after clinical onset (Figure 1). For the Euroimmun Zika virus IgM test, maximum percentage detection (71%) was 15–30 days after the onset of disease. After this time, the percentage of detectable IgM decreased rapidly, to only 21% of samples collected in days 31–60 and <9% for those collected >60 days after infection (Figure 1, panel A). The Dia. Pro Zika virus IgM test was more sensitive, with higher rates of positive samples observed over a longer time: 29% positive samples on days 0–4 after clinical onset, increasing to a maximum of 93% positivity during days 15–30. The positivity rate decreased more slowly than with the Euroimmun test, and 29% of samples were still positive for IgM during days 181–300 after infection. For Zika virus IgG, both assays detected the antibody in >40% of samples collected during the acute phase of disease (days 0–4) and in 100% of samples collected during days 31–180 after onset of disease (Figure 1, panel B). A slight decrease in the positivity rate for IgG observed with the Euroimmun assay before day 300 suggests a possible lack of sensitivity over time.

**Figure 2:**
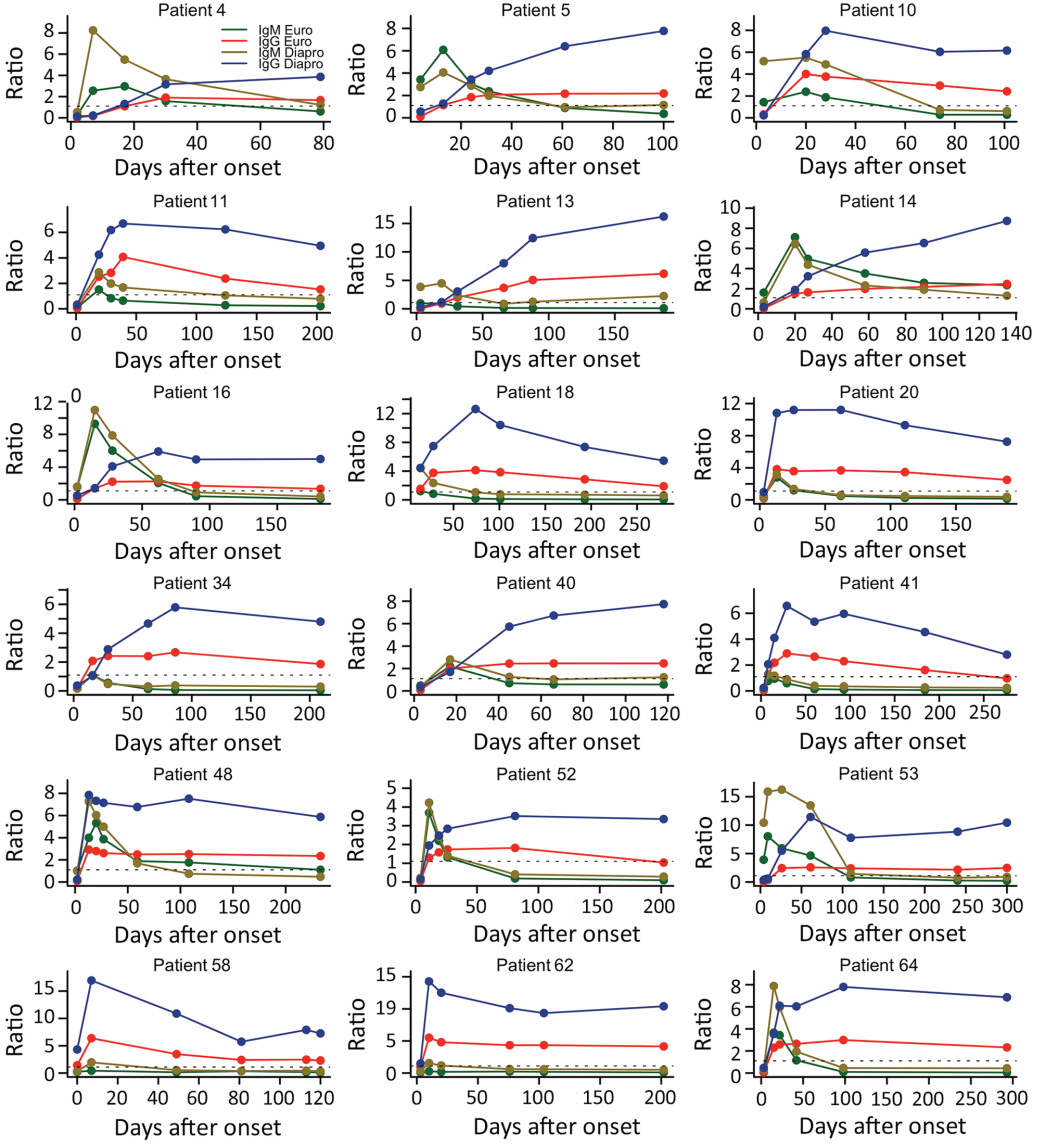
Individual time-course analyses of Zika virus IgM and IgG signal-to-cutoff ratios obtained by using Euroimmun and Dia.Pro kits for 18 patients for whom 5 or more sequential samples were available.

We also evaluated the evolution of the overall mean S/CO ratios by time and the test used (Figure 1, panels C and D). We observed similar kinetics for the mean S/CO ratios for the 2 assays: the maximum mean S/CO ratio peaked during days 15–31 for the IgM assays and during days 91–180 for the IgG assays. Nevertheless, the differences between the mean S/CO ratios for both the IgM and the IgG assays at each time after infection class were significant (all p<0.05) (Figure 1, panels C and D). The Dia.Pro assays gave higher S/CO ratios for the same threshold values, explaining the greater sensitivity of these tests. We obtained the kinetics of Zika virus IgM and IgG with both assays for patients for whom we had >5 sequential samples are (Figure 2).

## Discussion

In this assessment of the performance of Euroimmun and Dia.Pro Zika virus IgM and IgG ELISAs for diagnosis of Zika virus infection, we had a large panel of well-characterized samples from areas endemic for arboviruses, the dates of symptom onset, and infection confirmed by real-time RT-PCR. Although the Dia.Pro IgM assay was more sensitive (69%) than the Euroimmun IgM test (49%), the performance of both IgM assays was suboptimal. The sensitivity of a screening test is a major factor in determining its usefulness, because poor sensitivity of the first test used in a diagnostic algorithm could lead to false-negative results that would not be further evaluated.

The sensitivity of the Euroimmun Zika virus IgM test was significantly lower than that reported previously (*15**–**18*)*.* The differences might be caused by differences in study design, selection criteria, and the few positive samples in the previous studies (*17**–**18*). The lower sensitivity we found for this assay might also be the result of the larger proportion of secondary flavivirus infections in the panel used, as the samples were taken in an area endemic–epidemic for dengue and with mandatory vaccination against yellow fever. A significant lower sensitivity of the Euroimmun Zika virus IgM assay has also been reported in travelers from Israel compared with travelers from Europe and Chile, possibly related to the West Nile virus background immunity of the population of Israel (*19*). More recent evaluations have also reported low sensitivity (39.5% and 37%) of the Euroimmun assays (*20**,**21*)*.*

Sensitivity is essential for a frontline diagnostic test, and specificity should also be carefully evaluated. In our study, we assessed the specificity of all the tests with various non–Zika virus samples to evaluate potential cross-reactivity. A first subpanel of samples from patients with confirmed acute dengue virus infection was formed because of the high potential for flavivirus cross-reactivity; a second subpanel consisted of samples from patients with confirmed chikungunya virus infection; and a third subpanel consisted of samples from patients with no dengue virus, chikungunya virus, or Zika virus infection. The specificity of the Euroimmun Zika virus IgM assay was 99% and that of the Dia.Pro test was 96%, with cross-reactivity varying according to the subpanel. Most cross-reactions were observed in the subpanel of acute dengue samples, in which 3 of 33 samples were false positive for Zika virus IgM, whereas 1 of 74 samples collected >1 year after the dengue epidemic (chikungunya virus subpanel and dengue virus, chikungunya virus, and Zika virus negative subpanel) was false positive. Maximum cross-reactivity of IgG tests was seen in the subpanel collected in the post–dengue epidemic period in 2014–2015. The false-positivity rate was 40% (14/35) in the acute dengue subpanel for both commercial tests; the rate grew to 45.8% (11/24) for the Euroimmun Zika virus IgG test and 58.6% (17/29) for the Dia.Pro Zika virus IgG test in the subpanel of acute chikungunya virus samples collected right after the dengue epidemic period in 2014–2015. The IgG cross-reactions tended to decrease with time: 2 years after the end of the latest dengue epidemic in French Guiana, 5/42 (11.9%) samples were falsely positive by the Euroimmun IgG test and 9/42 (21.4%) samples were falsely positive with the Dia.Pro test. All samples except 2 with a false-positive result for Zika virus IgG were positive for dengue virus IgG by our in-house IgG antibody capture ELISA technique. The predictive positive value of both Zika virus IgG assays therefore largely depends on the epidemiologic situation of other flaviviruses, like dengue virus.

A study performed in Martinique during March–June 2016 during the Zika virus epidemic showed a good correlation between a high Euroimmun Zika virus IgG ratio and a positive Zika virus seroneutralization result. Ratios >4 were associated with positive seroneutralization in >95% of cases, whereas ratios >5 were associated with seroneutralization in 100% of cases (*22*)*.* At the time of the study, only sporadic dengue cases were reported in Martinique, as the previous epidemic occurred in 2013–2014, >2 years earlier. In our assessment, the IgG S/C ratio value of false Zika virus IgG–positive samples varied from 1.1 to 6.7 (mean 3.9) for acute dengue samples to 1.2 to 3.7 (mean 2.0) for other non–Zika virus samples. These results underline the importance and potential efficacy of using selected panels to evaluate performance and, especially, the specificity of serologic assays. These results also indicate that when there is major cocirculation of dengue virus and Zika virus, the interpretation of serologic assays could be increasingly complex.

The overall specificity of the Euroimmun Zika virus IgG assay was 79% and that of the Dia.Pro assay was 62%. These results indicate suboptimal specificity, which is lower than that reported previously (*20**,**21*), and is a concern for serologic diagnosis of Zika virus infection. Seroneutralization is the classical reference test for confirming contact with Zika virus in cases of positive results with ELISA assays in regions where flaviviruses cocirculate; however, even seroneutralization tests could be difficult to interpret, leading the US Centers for Disease Control and Prevention to change its guidance for interpretation of Zika virus antibody test results in May 2016 (*23**,**24*). In cases of secondary flavivirus infection, a microneutralization test might not discriminate between the past and recent infecting viruses, leading to an assumption of just a recent flavivirus exposure.

As reported previously by others, when IgM and IgG results were combined, sensitivity increased to 82% for Euroimmun and 87% for Dia.Pro assays, whereas specificity decreased to 69% for Euroimmun and 62% for Dia.Pro (*20**,**21*)*.* However, according to this combined analysis, 42% (30/71) of samples positive by the Euroimmun assays and 21% (16/75) for the Dia.Pro assays correspond to IgM negative/IgG positive samples, for which distinction between recent and past infections is not possible. Thus, a combined interpretation is not suitable for Zika infection diagnosis in the context of endemic–epidemic circulation.

We not only assessed the performance of Euroimmun and Dia.Pro Zika virus IgM and IgG tests but also evaluated the kinetics of the antibodies through 300 days after the onset of symptoms. A major concern in serologic diagnosis of Zika virus infection is determining the date of infection, due to the high proportion of asymptomatic forms. Data on the duration of IgM persistence after Zika virus infection are still limited, but our results indicate that this antibody could persist for at least several months, as described for other arboviruses (*25*)*.* Such persistence could preclude determination of the recent nature of an infection, and other assays should be evaluated. The analysis of individual Zika virus antibody kinetics revealed distinct patterns. In some patients (such as patients 4, 5, 10, 16, 53), high IgM ratios during the acute phase were associated with delayed and moderate increases in Zika virus IgG ratios, possibly reflecting a primary flavivirus or Zika virus infection; for other patients (such as patients 20, 34, 41, 58, 62), an early increase in the IgG ratio was combined with a low or even negative Zika virus IgM ratio throughout follow-up, indicating secondary flavivirus infections. These 2 types of kinetics showed contrasted signal intensities, which are not observed with our in-house ELISA assays, in which whole Zika virus is used as an antigen (data not shown).

A limitation of our study is that the Zika virus–positive samples were confirmed by RT-PCR and thus were all from symptomatic cases. If antibody levels are different in symptomatic and asymptomatic infections, as described for dengue, the performance and antibody kinetics observed in this study might be considerably different in samples from asymptomatic infections (*26**,**27*).

This study highlights the complexity of interpreting serologic assays in areas where various arboviruses cocirculate and demonstrates the importance of evaluating serologic assays with serum specimens from persons living in endemic–epidemic areas and use of parallel testing antibodies to maximize the reliability of diagnosis. Further studies are also needed to identify specific biomarkers of each flavivirus infection for diagnosis after the acute phase of disease.
